# Cationic Biomimetic Particles of Polystyrene/Cationic Bilayer/Gramicidin for Optimal Bactericidal Activity

**DOI:** 10.3390/nano7120422

**Published:** 2017-12-02

**Authors:** Gabriel R. S. Xavier, Ana M. Carmona-Ribeiro

**Affiliations:** Biocolloids Laboratory, Instituto de Química, Universidade de São Paulo, Av. Lineu Prestes 748, São Paulo 05508-000, SP, Brazil; gabriel.robert.xavier@usp.br

**Keywords:** polystyrene sulfate, dioctadecyldimethylammonium bromide, cationic bilayer fragments, gramicidin D, nanostructured cationic particles, optimization of bactericidal activity, *Escherichia coli*, *Staphylococcus aureus*

## Abstract

Nanostructured particles of polystyrene sulfate (PSS) covered by a cationic lipid bilayer of dioctadecyldimethylammonium bromide (DODAB) incorporated gramicidin D (Gr) yielding optimal and broadened bactericidal activity against both *Escherichia coli* and *Staphylococcus aureus*. The adsorption of DODAB/Gr bilayer onto PSS nanoparticles (NPs) increased the zeta-average diameter by 8–10 nm, changed the zeta-potential of the NPs from negative to positive, and yielded a narrow size distributions for the PSS/DODAB/Gr NPs, which displayed broad and maximal microbicidal activity at very small concentrations of the antimicrobials, namely, 0.057 and 0.0057 mM DODAB and Gr, respectively. The results emphasized the advantages of highly-organized, nanostructured, and cationic particles to achieve hybrid combinations of antimicrobials with broad spectrum activity at considerably reduced DODAB and Gr concentrations.

## 1. Introduction

Antimicrobial biomimetics encompass a very large variety of nanoparticles (NPs), most of them built by the assembly of different and/or similar molecules [1,2]. Many constructions evolved based on the assembly of quaternary ammonium cationic lipids [3,4,5], surfactants [4], polymers [6], antimicrobial peptides [7], and antibiotics with appropriate carriers [8]. Combinations of the active antimicrobials with inert materials, such as acrylates [9], polystyrene [10,11], carboxymethylcellulose [12], or lipids [13,14] improved the use and, eventually, the therapeutic index of old antimicrobials with novel formulations. In this era of antimicrobial resistance to antibiotics, it is important to improve the delivery of available antimicrobials via mechanical mechanisms of bacterial death able to circumvent the acquired resistance based on antibiotic chemical structures [15]. In this respect, cationic nanoparticles are of paramount importance since they mechanically attach to the microorganisms and often disassemble their cell wall, further penetrating and disrupting their cell membrane [12,16]. 

**Figure 1:**
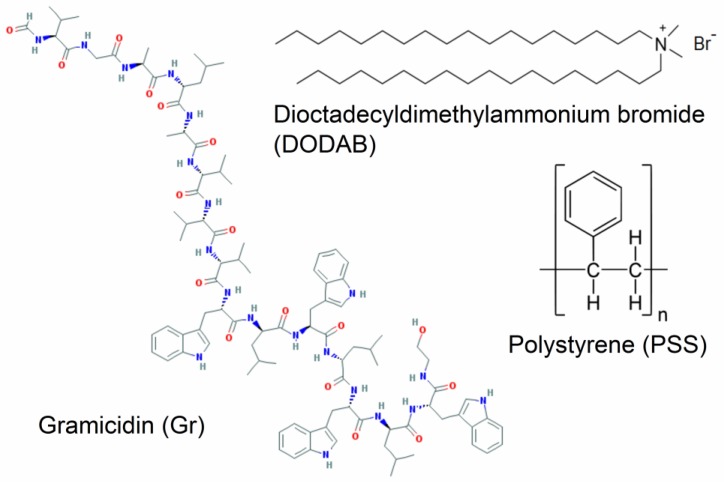
Chemical structure of the antimicrobial peptide Gr, the antimicrobial lipid DODAB, and the polymer polystyrene sulfate (PSS) used to build the cationic biomimetic NPs evaluated in this work regarding their antimicrobial properties. The industrial process for obtaining PSS employs potassium peroxydisulfate (KPS) as initiator for the polymerization of styrene. In water solution, KPS dissociates to give sulfate radicals. The sulfate radical adds to the alkene moiety of styrene forming the radical sulfate ester ●CHPhCH_2_OSO_3_^-^ that adds further alkenes via formation of C–C bonds to yield PSS. Thus, the sulfate moieties are covalently linked to polystyrene in PSS at the polymer chain terminus.

On the other hand, hybrid polymer-lipid NPs often exhibit a biomimetic character represented by a polymer core surrounded by a lipid bilayer [10,17,18,19,20]. The cationic bilayers supported on polystyrene sulfate nanospheres were previously described [10] and used to present antigens to the immune system for vaccines [21,22] or to compact giant DNA mimicking the histones [11]. The polymeric polystyrene sulfate (PSS) NPs are useful because all of them are nanosized, very homodisperse, and anionic-acting as model colloids able to become functional as antimicrobials from surface changes, such as dioctadecyldimethylammonium bromide (DODAB) bilayer adsorption or DODAB bilayer/gramicidin D (Gr) adsorption. In this work, we describe the antimicrobial application for the cationic biomimetic NPs of PSS/DODAB [10,11,21,22] and the incorporation of the very potent, but toxic, antimicrobial peptide Gr to build novel PSS/DODAB/Gr NPs with optimal antimicrobial activity at low DODAB and Gr doses, plus a broadened antimicrobial spectrum. Figure 1 shows the chemical structures of Gr, DODAB and the polystyrene polymer.

**Figure 2:**
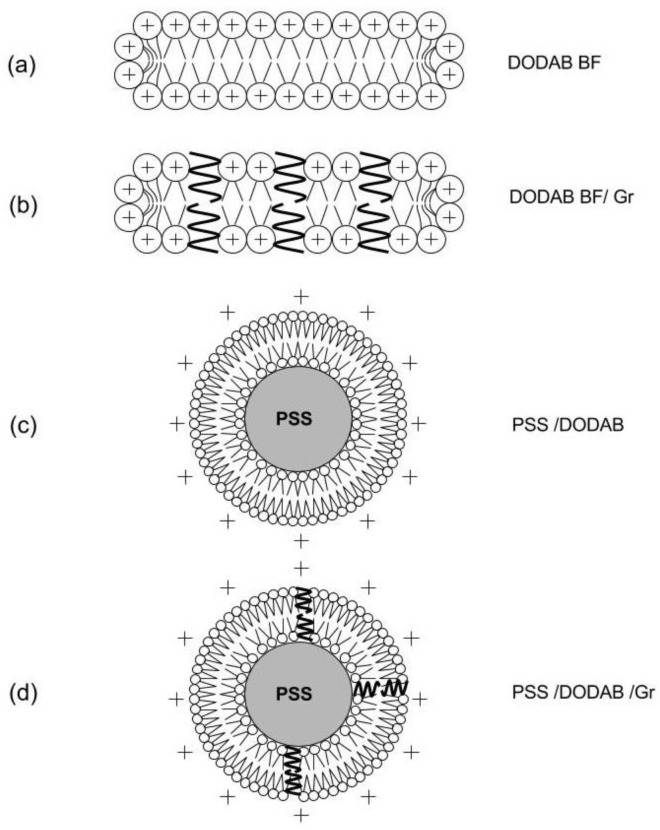
Procedure for obtaining the cationic biomimetic particles with two antimicrobials: DODAB and Gr dimeric channels. (**a**) Bilayer fragments (BF) of the cationic lipid DODAB [23,27]; (**b**) BF incorporating dimeric channels of Gr [24,25]; (**c**) PSS NPs covered by a DODAB bilayer [10]; and (**d**) PSS NPs covered by a DODAB bilayer with inserted Gr dimeric channels (this work). Schematic representations are cross-sections.

Figure 2 shows the cross-sections of the nanostructures obtained from the self-assembly of DODAB molecules dispersed by sonication with a macrotip as bilayer fragments (BF) [23], which were used to coat the anionic PSS NPs as such, or after incorporating the peptide Gr as dimeric channels [24,25,26].

## 2. Results

### 2.1. Physical Properties of the Dispersions

**Figure 3:**
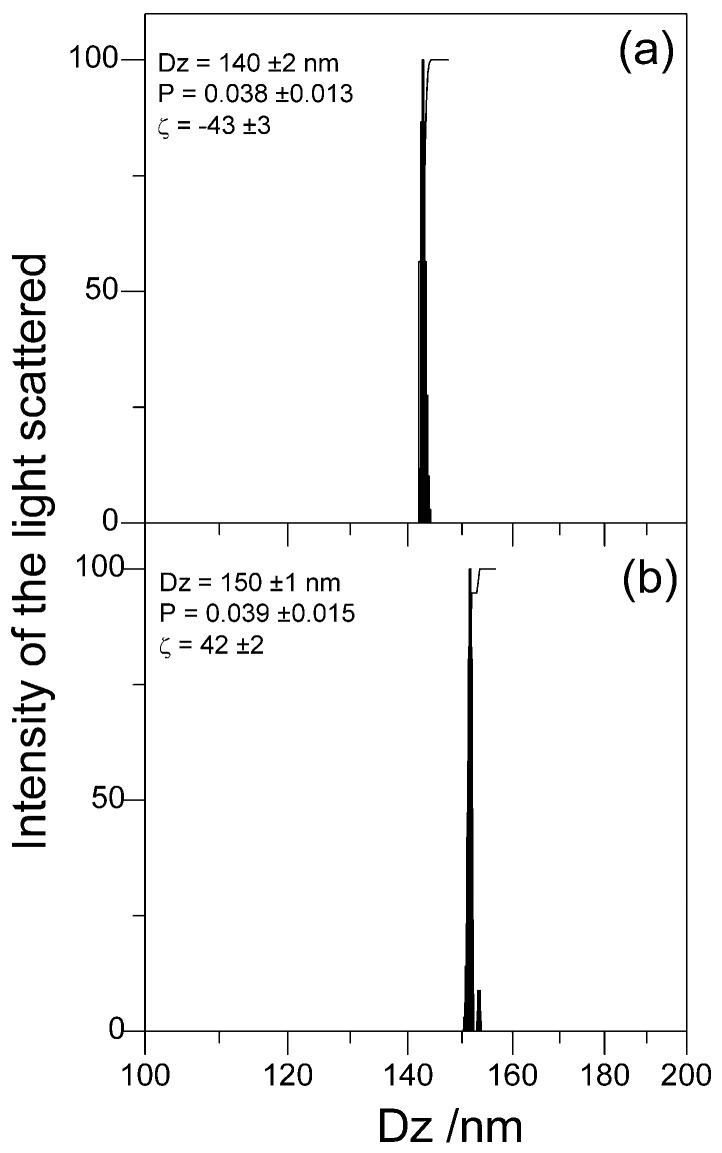
The narrow size distributions of the PSS based NPs used in this work as antimicrobials. (**a**) Size distributions for PSS NPs; (**b**) size distributions for PSS/DODAB/Gr NPs. Physical properties for the NPs are quoted in each subfigure: mean zeta-average diameter (Dz), polydispersity (P), and zeta-potential (ζ). PSS NP and PSS/DODAB/Gr NP concentrations were 2.4 × 10^10^ particles/mL, whereas [DODAB] and [Gr] were 0.01 and 0.001 mM, respectively.

In this section, the physical properties of the dispersions containing the PSS/DODAB/Gr NPs evidence their excellent colloidal stability undisturbed by the insertion of the Gr dimeric channels in the bilayer coating on PSS particles (Table 1). The main properties. such as size, zeta-potential, and polydispersity for the cationic biomimetic PSS/DODAB NPs, with or without Gr, revealed their model colloid nature with very narrow size distributions (low polydispersities), remaining as such both before and after inserting Gr in the DODAB bilayer coverage (Table 1, Figure 3). The comparison with data from the literature revealed the reproducibility of the physical properties of PSS/DODAB NPs that were very similar to those reported previously [11,21]. The same occurred for DODAB BF and DODAB BF/Gr with sizes, zeta-potentials, and polydispersities similar to those previously described [24]. Table 1 shows also that Gr insertion in the DODAB bilayers increased their zeta-potentials from 36–43 ± 2 mV up to 58–72 ± 2 mV. For the PSS/DODAB/Gr NPs the zeta-potential of 42 ± 2 mV was also higher than the value of 30 ± 2 mV for the PSS/DODAB NPs. The presence of Gr in the DODAB bilayer definitely increased the surface charge density on the NPs, which became more positive. Thus, the colloid stability of the dispersions improved due to the increased zeta-potential imparted by Gr incorporation though one cannot dismiss a possible role for the tryptophans anchoring Gr at the bilayer-water interface, which would represent some steric hindrance that would prevent the approach and aggregation of PSS/DODAB/Gr NPs. Both the coverage of PSS with a DODAB bilayer yielding PSS/DODAB and the coating of PSS NPs with a DODAB/Gr bilayer increased the mean hydrodynamic diameter (Dz) from 137–140 ± 1 nm up to 149–150 ± 1 nm as expected from the deposition of an 8–10 nm continuous bilayer onto each PSS NP in the dispersion. 

### 2.2. Microbicidal Activity of the Cationic Biomimetic NPs

**Figure 4:**
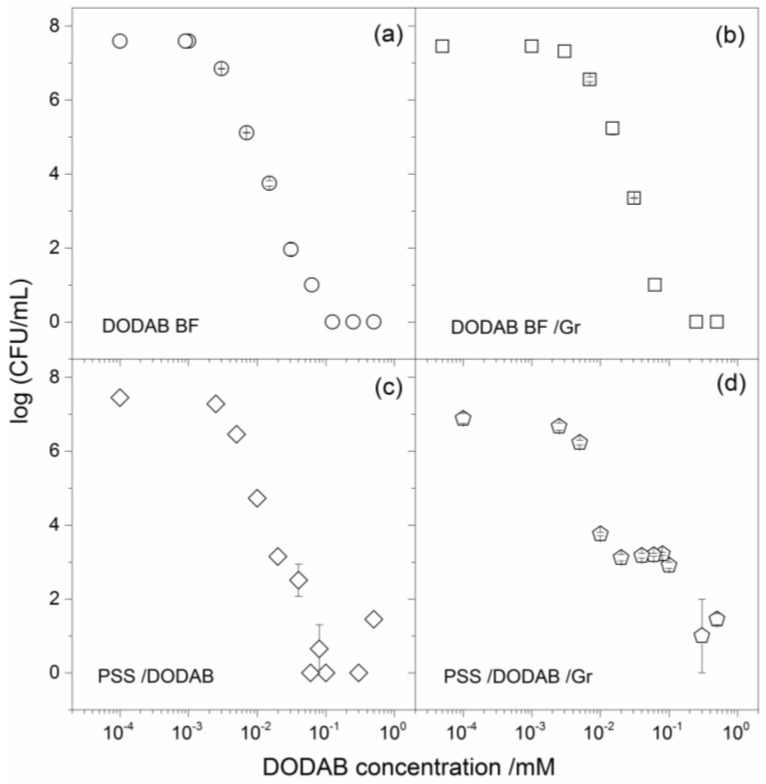
The microbicidal activity of biomimetic cationic NPs of PSS/DODAB and PSS/DODAB/Gr against *E. coli*. Cell viability of *E. coli*, in log (CFU/mL), as a function of DODAB concentration for different DODAB assemblies: (**a**) DODAB BF; (**b**) DODAB BF/Gr where Gr concentration is 10% of the DODAB concentration; (**c**) PSS/DODAB; (**d**) PSS/DODAB/Gr where Gr concentration is 10% of the DODAB concentration. Initial cell concentration was in the range (1–4) × 10^7^ CFU/mL. The ratio between DODAB concentration and NPs concentration was 10 µM DODAB per 2.4 × 10^10^ PSS particles/mL. DODAB and Gr concentration varied with the particle number density in (c) and (d). After 1 h interaction between cells and assemblies, dilution and plating of the mixtures allowed CFU counting.

**Figure 5:**
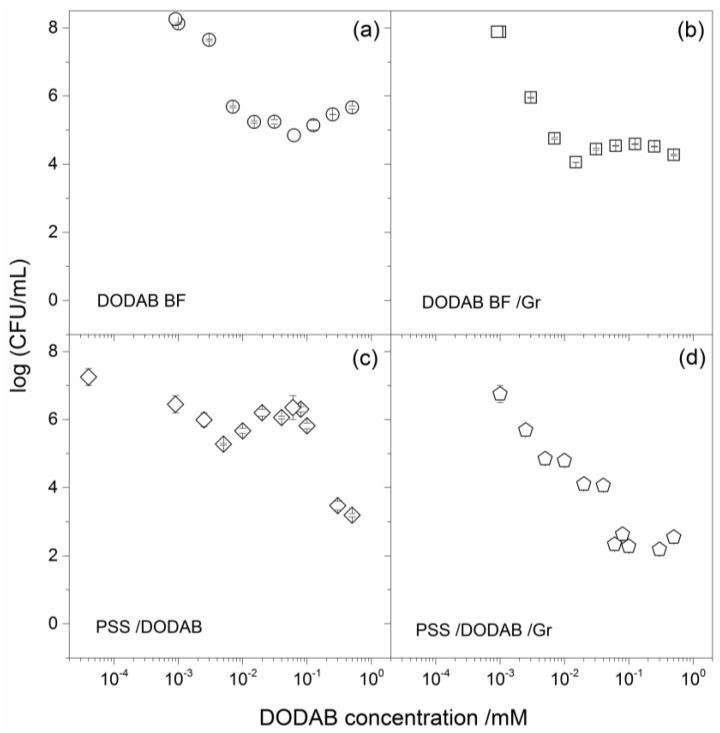
The microbicidal activity of biomimetic cationic NPs of PSS/DODAB and PSS/DODAB/Gr against *S. aureus*. Cell viability of *S. aureus*, in log (CFU/mL), as a function of DODAB concentration for different DODAB assemblies: (**a**) DODAB BF; (**b)** DODAB BF/Gr. where Gr concentration is 10% of [DODAB]; (**c**) PSS/DODAB; (**d**) PSS/DODAB/Gr. where Gr concentration is 10% of [DODAB]. Initial cell concentration was in the range (2–20) × 10^7^ CFU/mL. The ratio between DODAB and NP concentration was 10 µM DODAB per 2.4 × 10^10^ PSS particles/mL in (**c**) and (**d**). DODAB and Gr concentration varied with the particle number density in (**c**) and (**d**). After 1 h of interaction between cells and assemblies, dilution and plating of the mixtures allowed CFU counting.

**Figure 6:**
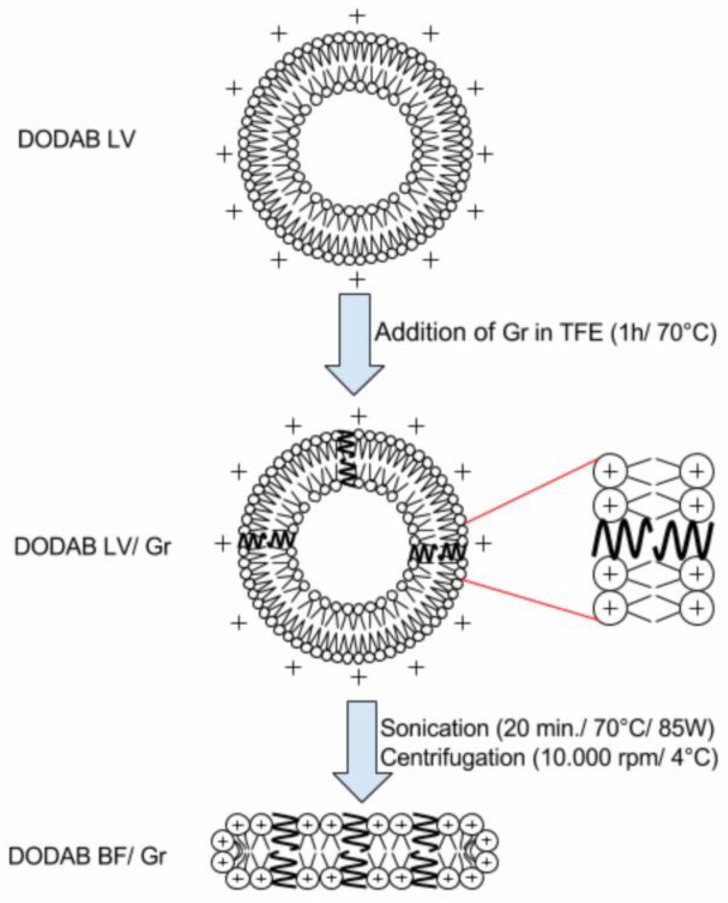
Procedure for obtaining Gr dimeric channels inserted in DODAB BF. The preparation of the DODAB BF/Gr assemblies from large DODAB vesicles (DODAB LV) followed a previously described procedure [24]. These BF/Gr nanostructures interacted with the PSS NPs to yield the cationic biomimetic particles with the microbicidal properties described in this work.

PSS/DODAB and PSS/DODAB Gr exhibited microbicidal activity, as shown from the systematic evaluation of microbial cell viability (log (CFU/mL)) as a function of the NPs’ concentration expressed as DODAB concentration in Figure 4 and Figure 5. The cell viability of representative pathogenic bacteria, such as *Escherichia coli* and *Staphylococcus aureus*, displayed a clear dependence on the concentration of DODAB (Figure 4 and Figure 5). One should notice that Gr concentrations always corresponded to 10% of DODAB molar concentrations in Figure 4d and Figure 6d. The NPs, over a range of concentrations, yielded the DODAB concentrations shown on the cell viability curves in Figure 4c,d and Figure 5c,d. The molar concentration of DODAB required to cover all NPs with a cationic bilayer was easily calculated. The total surface area for PSS NPs with 137 nm mean diameter at 2.4 × 10^10^ particles/mL, the area per DODAB molecule at the air-water interface was equal to 0.6 nm^2^ [28] and the assumption that, on each bilayer-covered NP, two DODAB molecules occupy 0.6 nm^2^ due to bilayer adsorption, allowed obtaining the [DODAB] for bilayer-covered NPs. Thus, 0.0078 mM of DODAB covered 2.4 × 10^10^ particles per mL with one bilayer. Considering some DODAB adsorption on the container surfaces, in order to cover all NPs with a DODAB bilayer, 0.0100 mM DODAB interacted with NPs at the quoted concentration for 1 h at room temperature before proceeding with the determinations of antimicrobial activity. In order to vary the concentration of the antimicrobials, DODAB, and Gr against the microorganisms, the NP concentration varied at a constant ratio between surface areas for NPs and DODAB in DODAB BF: 0.01 mM DODAB per 2.4 × 10^10^ particles/mL.

The cell viability curves provided detailed information on minimal bactericidal concentrations (MBC) and the extent of the microbicidal activity given by the total reduction of CFU counting expressed as log (CFU/mL) at the MBC (Figure 4, Figure 5, and Table 2). 

The results in Table 2 showed a reduction of about 8 log (CFU/mL) in the viability of *E. coli* caused by DODAB BF (Figure 4a), which was very similar to the one caused by PSS/DODAB (Figure 4c). Consistently, at MBC values for DODAB BF and PSS/DODAB, there was a viability reduction of about 7.5–7.6 log (CFU/mL) for both dispersions (Table 2). The insertion of Gr in the supported DODAB bilayer (PSS/DODAB) reduced the excellent DODAB BF and PSS/DODAB activity against *E. coli*. Possibly, some steric hindrance due to Gr in the PSS/DODAB NPs hampered the interaction and delivery of the NPs to the cells. In fact, the absence of Gr activity against *E. coli* was previously reported [24,25]. The NPs concentration of PSS/DODAB/Gr was substantially increased in order to increase DODAB concentration and thereby reduce the *E. coli* cells viability of about 6 log (CFU/mL) (Table 2).

Against *S. aureus* DODAB was less effective than against *E. coli*, as depicted from the large NP concentrations required for PSS/DODAB in order to achieve a reduction of about 4.6 log (CFU/mL) in cell viability (Table 2). The combinations with Gr were more successful against *S. aureus* allowing to achieve a reduction in cell viability of about 6 log (CFU/mL) for PSS/DODAB Gr NPs at 0.057 mM DODAB and 0.0057 mM Gr (Table 2). Important conclusions can be drawn from the comparison between the four different assemblies. Among the assemblies tested, the PSS/DODAB/Gr NPs reached the most potent microbicidal effect against *S. aureus.* Importantly, at 0.057 mM DODAB in these NPs, this combination would also be very effective against *E. coli* reducing the log (CFU/mL) by 7.5 log (CFU/mL). Thus, at the tiny doses of 0.057 mM DODAB and 0.0057 mM Gr were very well organized around the PSS NPs, and optimal and broadened activity against both bacteria was achieved. The PSS/DODAB/Gr NPs displayed maximal microbicidal activity at minimal doses of the toxic antimicrobials: the cationic lipid and the antimicrobial peptide.

## 3. Discussion

The biomimetic organization built in this work provided optimal functionality for combinations of two different antimicrobials: the cationic lipid DODAB, which preferentially kills Gram-negative bacteria, and the neutral antimicrobial peptide Gr, which preferentially kills Gram-positive bacteria. This combination in particular broadened the spectrum of antimicrobial activity assuring low MBC values for each component in the combination and possibly reducing dose-associated toxicity. The cationic biomimetic NPs of PSS/DODAB/Gr may find interesting applications in biomedical devices, such as antimicrobial coatings, burns and diabetes wounds, and ulcers, implants, and dentistry materials [7,29,30]. Food technology might also benefit from biopolymer films embedded with the cationic antimicrobial particles described here [24,31]. 

The lipid bilayer adsorbed to the oppositely-charged PSS NP imparted the biomimetic character to the polymeric nanoparticles that allowed optimal insertion of the peptide as dimeric channels as shown before from circular dichroism spectra and loss of osmotic responsivity for closed, large DODAB vesicles due to Gr dimeric channel insertion in the DODAB bilayer [25,26]. In [25,26], circular dichroism (CD) and fluorescence spectroscopy, plus the lack of osmotic response of large vesicles (LV)/Gr, revealed the fact that Gr dimeric channels were found in the DODAB LV. CD and intrinsic fluorescence spectra similar to those in trifluoroethanol (TFE) and KCl or glucose permeation through the LV/Gr bilayer revealed the Gr dimeric channel conformation. For Gr in BF the intertwined dimeric, non-channel conformation was also depicted from CD and intrinsic fluorescence spectra similar to those for Gr in ethanol [25,26]. The higher surface charge density of PSS/DODAB/Gr NPs, in comparison to PSS/DODAB NPs, inferred from the higher zeta potentials for PSS/DODAB/Gr, as compared to those of PSS/DODAB (Table 1), also evidenced Gr insertion in the bilayer and compression of charged polar heads that increased the surface charge density of the supported cationic bilayers on the NPs. Bilayer adsorption of phospholipids functionalized with the arginil-glycil-aspartil or Arg-Gly-Asp (RGD ) peptide on mesoporous silica particles embedded with arsenic trioxide improved the delivery of this anticancer drug to hepatic carcinomas [32]. The utility of the biomimetic NPs, however, is not restricted to drug delivery; many other uses can be foreseen for disinfection and sterilization, for example, in catheters and probes, wound dressings, and antimicrobial therapy against antibiotic-resistant strains. In particular, latex particles find many applications in the painting industry and the cationic biomimetic NPs could be part of useful antimicrobial paints. 

The relevant physical properties for the PSS/DODAB/Gr NPs were: (1) the positive charge that drove them to any oppositely-charged cell or surface [33,34]; (2) the highly-organized outer layer on the NP prone to deliver both the antimicrobial lipid and the peptide to the bacterial cells at reduced and less toxic doses of both (Figure 4 and Figure 5; Table 2); (3) the possibility of building films and coatings with the NPs on surfaces in order to fight the formation of biofilms [4,15,35].

In this work the procedure for reconstitution of functional Gr dimeric channels involved the preliminary insertion of Gr in the LV bilayers of closed and large DODAB vesicles (LV), which was followed by LV disruption by sonication to yield DODAB BF/Gr (Figure 2). This procedure was necessary because Gr previously displayed high affinity for pre-formed bilayer fragments where Gr conformation turned out to be the non-channel, intertwined dimers at the borders of the bilayer fragments [25,26]. The ionic channels of gramicidin D acquired their optimal conformation as such in the DODAB BF obtained from LV/Gr by ultrasonic disruption [24]. Gr concentration in lipid dispersions before and after filtering was estimated from Gr intrinsic fluorescence spectra by determining the total area under the spectra [25,26]. These experiments revealed the Gr preference for following the lipids. When the lipids of LVs were retained by the filtration membrane, Gr was also retained. When the lipids of BFs were in the filtrate, Gr was, too. Therefore, Gr molecules were not in the water solution; instead they were found embedded in the lipid bilayer in the form of functional dimeric channels.

The compatibility of DODAB with certain polymers, such as the acrylates, opened the possibility of embedding the cationic antimicrobial lipid in the polymeric network of polymer films in the absence of lipid bilayers; in this case the coatings killed bacteria upon contact without DODAB diffusion to the outer medium [36]. Otherwise the electrostatic attraction between anionic surfaces of flat silicon wafers [34] or silica nanoparticles yielded adsorption of cationic DODAB bilayers also useful as biomimetic antimicrobial coatings [5]. Although polystyrene and DODAB in spin-coated films did not result in hybrid materials due to phase separation, in the case of the polystyrene sulfate NPs the electrostatic attraction, similarly to silicon wafers or silica NPs, drove the bilayer adsorption and the formation of biomimetic NPs [10]. These cationic biomimetic NPs were first described in the early 1990s [10], but had not been evaluated before as antimicrobial nanomaterials. This work showed that DODAB and Gr as antimicrobials incorporated in the bilayer that surrounded the polymeric NP allowed advantageous optimization and broadening of the antimicrobial activity. From Figure 4 and Figure 5, the bactericidal activities of DODAB or PSS/DODAB (due to the quaternary ammonium nitrogen in the polar head of the DODAB molecule) were broadened for the PSS/DODAB/Gr systems. Gr added the disturbance of the ionic balance in the bacterial cells due to the presence of Gr dimeric channels, which were possibly transferred to the bacterial cells allowing to extend the activity spectrum to optimize the killing of *S. aureus*. 

## 4. Materials and Methods

### 4.1. Materials

DODAB, gramicidin D, 2,2,2-trifluoroetanol (TFE), and sodium chloride (NaCl) were from Sigma-Aldrich (St Louis, MO, USA). Anionic polystyrene sulfate (PSS) NPs (lot no. 10-307-57), nominal mean diameter of 0.137 ± 0.003 µm, 415,124 cm^2^/g of specific surface area (SSA), surface charge density of 0.79 µC/cm^2^, and –43 ± 3 mV of zeta-potential (Table 1) were obtained from Interfacial Dynamics Corporation (Portland, OR, USA) and the stock suspension containing 5.97 × 10^13^ particles/mL was further diluted in 1 mM NaCl solution in order to obtain the final desired NP concentration. The chemical structures of the components of the biomimetic NPs are shown on Figure 1. 

### 4.2. Preparation of the Lipid Dispersions

DODAB dispersions were prepared as previously described [25]. DODAB LV were obtained by hydration and vortexing of the DODAB powder in 1 mM NaCl at 60 °C, a temperature arbitrarily chosen, which was above the main gel to liquid-crystalline phase transition temperature (T_m_) of the DODAB bilayer, namely, above 47–49 °C [37], until complete dispersion of DODAB at 2 mM DODAB.

DODAB BF dispersions were prepared from DODAB LV from sonication with a macrotip (85 W/15 min/above T_m_) followed by centrifugation (9300× *g*/60 min/4 °C) to precipitate the titanium particles ejected by the macrotip during the sonication procedure. The final DODAB concentration in the dispersions was precisely determined by halide microtitration as previously described [38]. The ultrasonic disruption of the large vesicles (LV) yielded the DODAB BF. In order to obtain the peptide dimer inserted in the middle of the BF the hint was to prepare the BF/Gr from the LV/Gr by sonication of the latter [24]. This avoided the presence of intertwined Gr dimers at the borders of the BF [24,25]. 

### 4.3. Preparation of DODAB/Gr Dispersions

Aliquots of a stock solution of Gr (6.4 mM Gr in TFE) were added to pre-formed DODAB bilayers at 10:1 molar ratio DODAB:Gr before heating at 60 °C for 1 h [26]. The DODAB BF/Gr dispersions were prepared in a different manner than the one described in [25], namely, after preparation of LV/Gr, the dispersion was sonicated with a macrotip (85 W for 15 min at 70 °C) and centrifuged (93,000× *g* for 60 min at 4 °C). In [25], Gr had been added directly to pre-formed DODAB BF, yielding intertwined Gr molecules at the border of the bilayer fragments. In this work, the procedure was changed to disrupt the DODAB LV/Gr thereby optimizing the dimeric channel conformation of Gr in the DODAB BF/Gr dispersion thus obtained. Figure 6 shows the procedure to obtain Gr as dimeric channels inserted in the DODAB BF bilayer. DODAB large vesicles (LV) were previously found to incorporate the peptide Gr as functional dimeric channels [26,39]. In order to obtain DODAB BF with Gr dimeric channels, the LV were disrupted by sonication with a macrotip, yielding DODAB BF/dimeric channel Gr (Figure 6).

### 4.4. Preparation of PSS/DODAB and PSS/DODAB/Gr Dispersions

The PSS/DODAB dispersion was prepared as previously described [11]. With BF at 2 mM and PSS at 5.97 × 10^13^ particles/mL, both dispersions at 1 mM NaCl were diluted to the final desired concentration using this same NaCl solution. For example, 0.2 mL of PSS stock dispersion, 2.5 mL of DODAB BF at 2 mM DODAB, and 2.299 mL of 1 mM NaCl solution were mixed and interacted for 1 h at 25 °C yielding 2.4 × 10^12^ particles/mL and 1 mM DODAB. 

The PSS/DODAB/Gr dispersions were prepared similarly at the same final concentrations. 

PSS and DODAB BF or DODAB BF/Gr interacted in 1 mM NaCl for 1 h before performing their physical characterization.

### 4.5. Physical Characterization of the Dispersions by Dynamic Light Scattering (DLS)

The characterization of the PSS/DODAB or PSS/DODAB/Gr NPs was performed by means of dynamic light scattering (DLS) using a Brookhaven ZetaPlus-ZetaPotential Analyzer (Brookhaven Instruments Corp., Holtsville, NY, USA), equipped with a 677 nm laser and a correlator for DLS at 90° plus software for z-average diameter (Dz), zeta potential (ζ), and polydispersity (P) determinations and evaluation of the size distributions [40]. The zeta potential was obtained from the electrophoretic mobility μ and the Smoluchowski equation: ζ = μη/ε, where η is the medium viscosity and ε is the dielectric constant of the medium. PSS, PSS/DODAB, or PSS/ DODAB/Gr dispersions were diluted 1:20 before measurements. The Brookhaven apparatus algorithm calculated the size distributions, always attempting to obtain them as several peaks of the intensity of light scattered [40]. On the other hand, the log normal or Gaussian size distribution fitted the intensity of the light scattered to produce only one peak with a mean z-average diameter (Dz) value. The hydrodynamic radius was determined by the software of the Brookhaven apparatus, using a mathematically well-defined algorithm for determining the hydrodynamic radius and diameter from quasi-elastic light scattering [40]. The apparatus performed at least 10 repeats; usually, the mean Dz was obtained from 10 to 40 determinations. The size distributions, Dz, P, and ζ of the dispersions physically characterized the dispersions.

### 4.6. Bacterial Growth and Cell Viability from Plating and CFU Counting

Bacterial growth was performed as previously described for *E. coli* and *S. aureus* [12]. Briefly, the bacteria *E. coli* ATCC 25922 and *S. aureus* ATCC 29213 were obtained from The American Type Culture Collection (ATCC, Manassas, VA, USA). The lyophilized strains, kept at –20 °C, were incubated with Mueller Hinton (MHA) agar (37 °C/18–24h). Thereafter, a few isolated colonies from the agar plates were transferred to a 1 mM NaCl solution for adjusting the turbidity at 625 nm to 0.08–0.1, which is equivalent to 0.5 of the McFarland scale [41]. 

Bacterial cell viabilities were determined after 1 h interaction between the dispersions and the cells in 1 mM NaCl solutions. Thereafter, the mixtures were diluted up to 100.000 times in order to spread around 100 viable cells on the agar plates. The appropriate controls for cells alone and dispersions alone were performed. The agar plates with bacteria/dispersions aliquots plated in triplicate were incubated (37 °C/24–48h) and then counted for CFU counting. The mean cell viability was plotted as log (CFU/mL) as a function of DODAB concentration.

## 5. Conclusions

PSS/DODAB/Gr NPs reached the most potent microbicidal effect against *S. aureus.* Importantly, at 0.057 mM DODAB in these NPs, this combination was also very effective against *E. coli* reducing the log (CFU/mL) by 7.5 log (CFU/mL). Thus, at the very small doses of 0.057 mM DODAB and 0.0057 mM Gr organized very well around the PSS NPs, and optimal and broadened activity against both bacteria was achieved. The PSS/DODAB/Gr NPs displayed maximal microbicidal activity at minimal doses of the toxic antimicrobials: the cationic lipid and the antimicrobial peptide. The cationic biomimetic NPs based on polymeric and nano-sized PSS represent model antimicrobial colloids able to carry a variety of hydrophobic, hydrophilic, or amphiphilic antimicrobial substances in a very organized, biomimetic manner. These properties may lead to interesting biomedical, industrial, and pharmaceutical applications in the future. 

## Figures and Tables

**Table 1:** Physical characterization of DODAB BF, DODAB BF/Gr, PSS, PSS/DODAB, and PSS/DODAB/Gr assemblies in 1 mM NaCl aqueous solution.

Assembly	Dz (nm)	ζ (mV)	P	References
DODAB BF	59 ± 1	43 ± 2	0.215 ± 0.006	[24]
DODAB BF	55 ± 1	36 ± 2	0.248 ± 0.004	This work
DODAB BF/Gr	54 ± 1	72 ± 4	0.277 ± 0.004	[24]
DODAB BF/Gr	71 ± 1	58 ± 2	0.261 ± 0.003	This work
PSS	137 ± 2 *	-	-	-
PSS	140 ± 2	-43 ± 3	0.038 ± 0.013	This work
PSS/DODAB	149 ± 1	30 ± 2	0.049 ± 0.014	[11]
PSS/DODAB/Gr	150 ± 1	42 ± 2	0.039 ± 0.015	This work

* The NPs mean diameter was provided by the supplier from scanning electron micrographs by taking the mean value from 500 NPs.

**Table 2:** MBC, in mM, and maximal reduction of cell viability, in log (CFU/mL), for DODAB in different assemblies against *E. coli* and *S. aureus*.

Assembly	MBC (mM)/log (CFU/mL)	
	*E. coli*	*S. aureus*
DODAB BF	0.063/7.6	0.063/3.4
DODAB BF/Gr	0.031/7.5	0.015/3.8
PSS/DODAB	0.059/7.5	0.471/4.6
PSS/DODAB/Gr	0.300/6.0	0.057/5.7
Gr	0.010/0.3 *	0.010/2.1 *

* Data taken from [24,25].
